# Reduced Orexin System Function Contributes to Resilience to Repeated Social Stress

**DOI:** 10.1523/ENEURO.0273-17.2018

**Published:** 2018-04-16

**Authors:** Laura A. Grafe, Darrell Eacret, Jane Dobkin, Seema Bhatnagar

**Affiliations:** 1 Department of Anesthesiology and Critical Care, Children’s Hospital of Philadelphia, Philadelphia, Pennsylvania 19104; 2 Perelman School of Medicine, University of Pennsylvania, Philadelphia, Pennsylvania 19104

**Keywords:** Anxiety and depression, DREADDs, hypocretin, resilience, social defeat, vulnerability

## Abstract

Exposure to stress increases the risk of developing affective disorders such as depression and post-traumatic stress disorder (PTSD). However, these disorders occur in only a subset of individuals, those that are more vulnerable to the effects of stress, whereas others remain resilient. The coping style adopted to deal with the stressor, either passive or active coping, is related to vulnerability or resilience, respectively. Important neural substrates that mediate responses to a stressor are the orexins. These neuropeptides are altered in the cerebrospinal fluid of patients with stress-related illnesses such as depression and PTSD. The present experiments used a rodent social defeat model that generates actively coping rats and passively coping rats, which we have previously shown exhibit resilient and vulnerable profiles, respectively, to examine if orexins play a role in these stress-induced phenotypes. *In situ* radiolabeling and qPCR revealed that actively coping rats expressed significantly lower prepro-orexin mRNA compared with passively coping rats. This led to the hypothesis that lower levels of orexins contribute to resilience to repeated social stress. To test this hypothesis, rats first underwent 5 d of social defeat to establish active and passive coping phenotypes. Then, orexin neurons were inhibited before each social defeat for three additional days using designer receptors exclusively activated by designer drugs (DREADDs). Inhibition of orexins increased social interaction behavior and decreased depressive-like behavior in the vulnerable population of rats. Indeed, these data suggest that lowering orexins promoted resilience to social defeat and may be an important target for treatment of stress-related disorders.

## Significance Statement

Stress-related mental illnesses occur in only a subset of individuals, whereas others are resilient to the effects of stress. Our work used an animal model of social stress to identify a substrate of resilience, the neuropeptides orexins, which are known to be altered in patients with major depressive disorder and PTSD. We found that orexins are decreased in rats resilient to social stress. To test whether low orexins contribute to resilience, orexins were inhibited during 3 d of a social defeat stress paradigm, which increased subsequent social interaction behavior and decreased depressive-like behaviors in a previously vulnerable population of rats. This suggests that lowering orexins is important in promoting resilience to stress and that orexins are an important target for treatments of stress-related illness.

## Introduction

Exposure to chronic stress is associated with the onset and increased incidence of stress-related mental illness such as depression, anxiety-related disorders, and post-traumatic stress disorder (PTSD) (McEwen and Stellar, 1993; Yehuda et al., 1994; Ehlert et al., 2001). However, these disorders occur in only a subset of individuals that are more vulnerable to the effects of stress, whereas others remain resilient to the effects of stress. The neurobiological basis for these vulnerable and resilient phenotypes is not fully understood. Determining the neural substrates underlying vulnerability or resilience could lead to individualized treatment to either prevent vulnerability or promote resilience to stress.

Many stress-related disorders are associated with alterations in arousal. For example, PTSD is characterized by hypervigilance and hyperarousal to stimuli related to the traumatic event (Yehuda, 2000). Important neural substrates that mediate arousal, wakefulness, and vigilance are the neuropeptides orexins (de Lecea et al., 1998; Sakurai et al., 1998). Extending beyond their role in mediating general arousal and wakefulness, orexins are important in the response to stressful stimuli that require the animal to shift from a basal to a reactive state (Berridge and España, 2005). More specifically, orexins are known to promote the stress response including activation of both the sympathetic nervous system and the hypothalamic-pituitary-adrenal axis (Jászberényi et al., 2000; Kuru et al., 2000; Winsky-Sommerer et al., 2005; Spinazzi et al., 2006; Heydendael et al., 2011; Kuwaki, 2011; Johnson et al., 2012; Messina et al., 2014). Conversely, orexin neurons are activated by stressors such as forced swim and can also be activated by direct administration of the stress regulatory peptide corticotropin-releasing hormone (Winsky-Sommerer et al., 2005; Chang et al., 2007; Furlong et al., 2009; Chen et al., 2013). Importantly, orexin levels are altered in the CSF of patients with depression and PTSD. Together, both preclinical and clinical data suggest that orexins are involved in the processes by which stress leads to some psychiatric disorders (Strawn et al., 2010; Johnson et al., 2012). However, it is not known whether orexins contribute to individual differences that occur in response to stress, which are important in determining an individual’s resilience or vulnerability to some psychiatric disorders.

One factor relating to susceptibility and resiliency is the coping style adopted to deal with the stressor (Veenema et al., 2003). Both active coping, characterized by the fight or flight response, and passive coping, characterized by heightened immobility, could be engaged during exposure to threatening stimuli (i.e., stressors; Engel and Schmale, 1972; Koolhaas et al., 1999; Southwick et al., 2005; Wood and Bhatnagar, 2015). Clinical studies have indicated that humans demonstrating passive coping are more likely to develop depression than those who display active coping (Folkman and Lazarus, 1980; Billings and Moos, 1984). The present experiments used an animal model of social stress in which coping strategies vary and are associated with resilience or vulnerability to stress, as assessed by measures in the neuroendocrine system, behavior (Wood et al., 2010, 2015; Chen et al., 2015b; Finnell et al., 2017b), and inflammatory processes (Pearson-Leary et al., 2017).

These experiments aimed to examine orexins as a potential substrate underlying differences in vulnerability and resilience in response to social defeat stress in rats. First, orexin expression was measured by *in situ* radiolabeling and by quantitative PCR (qPCR) in passive coping (vulnerable) and active coping (resilient) rats, revealing that orexin expression was lower in resilient rats. This led to the hypothesis that lower levels of orexin underlie resilience to repeated social stress. To test this hypothesis, after rats had established active or passive coping phenotypes over 5 d of social defeat, orexin neurons were inhibited before each social defeat for three additional days using designer receptors exclusively activated by designer drugs (DREADDs). Dampening orexin action in passively coping rats before each defeat increased social interaction and decreased depressive-like behavior, promoting resilience. These studies establish that low orexin function contributes to the active/resilient behavioral phenotypes in response to repeated social defeat stress.

## Materials and Methods

### Animals

Adult, male Sprague-Dawley rats (275–300 g at time of stress) were used as controls or intruders (Charles River), and male Long-Evans retired breeders (650–850 g) served as residents (Charles River). Rats were individually housed with a 12-h light, 12-h dark cycle (lights on at 0700) in a climate-controlled room with *ad libitum* food and water. Rats were given 5 d of acclimation before experimentation. Studies were approved by Children’s Hospital of Philadelphia Research Institute’s Institutional Animal Care and Use Committee and conformed to the National Institutes of Health Guide for the Use of Laboratory Animals.

### Social defeat paradigm

**Figure 1.**
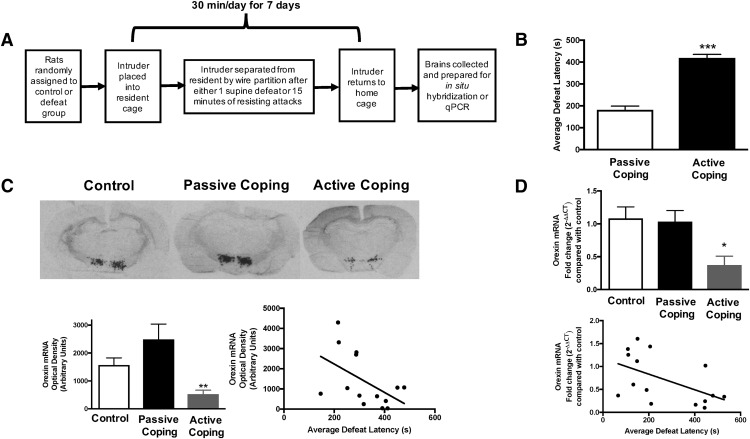
Social defeat paradigm and prepro-orexin expression in control and defeated rats. ***A***, Social defeat paradigm. ***B***, Average defeat latency over 7 d of social defeat. Passively coping rats have an average defeat latency <300 s, whereas actively coping rats have an average defeat latency >300 s. ***C***, Prepro-orexin expression in control, passive coping, and active coping rats. Top: representative images of *in situ* radiolabeling for prepro-orexin in control, passive coping, and active coping rats. Bottom: quantification of *in situ* radiolabeling in each treatment group reveals that actively coping rats have significantly less prepro-orexin mRNA in the lateral hypothalamus compared with control or passively coping rats. There is a negative correlation between average defeat latency and *in situ* radiolabeled orexin mRNA. ***D***, Prepro-orexin mRNA expression in control, passive coping, and active coping rats as measured by qPCR. Actively coping rats express significantly less prepro-orexin mRNA than control and passively coping rats. There is a negative correlation between average defeat latency and qPCR-quantified orexin mRNA. *, *p* < 0.05; **, *p* < 0.01; ***, *p* < 0.001.

The social defeat paradigm used in this study was based on the resident-intruder model originally developed by Miczek (1979) (see Fig. 1*A*). Sprague-Dawley rats were randomly assigned to either a control or social defeat group. During social defeat, each rat was placed into the home cage of an unfamiliar Long-Evans retired breeder (resident) for each of 5–8 consecutive days. Typically, the resident and intruder investigate each other for a short period of time (1–3 min), followed by attacks by the resident, which result in a defeat of the intruder. A defeat was determined when the intruder assumed a supine posture and froze for at least 2–3 s. On assuming the defeat posture, the resident and intruder were separated by a wire mesh barrier until 30 min had elapsed from time of initial placement into the cage of the resident. The barrier allowed for visual, auditory, and olfactory contact but prevented physical contact and further attacks on the intruder. The latency to be defeated was then recorded. If no defeat occurred within 15 min, the rats were separated with a wire mesh barrier for the remaining 15 min. Control rats were placed into a clean novel cage behind a wire mesh barrier for 30 min. Once the 30-min social stress was complete, each rat was placed back in its home cage. The average latency of each rat over the course of 7 d was entered into an R script used to perform cluster analyses on average defeat latencies (code available at www.github.com/cookpa/socialdefeat). The bootstrap classification starts from the assumption that the average latencies are drawn from a bimodal distribution. An initial classification of the average latencies is performed using “partitioning around medoids” (PAM) implemented in R’s cluster package (Reynolds et al., 1992). PAM is a robust implementation of *k*-means clustering, which separates data into a predefined number of clusters (in this case, 2, one for passive coping and one for active coping). The bootstrap algorithm resamples the data to assess the uncertainty in the classification. For each bootstrap iteration, we sample with replacement from the original latencies and rerun the PAM clustering. After 10,000 iterations, we define the probability of active coping classification for each of the average latencies as the fraction of the 10,000 bootstrap iterations in which that latency is classified as active coping. Latencies that are consistently classified as active coping have probability 1.0, and those classified consistently as passive coping have probability 0.0. Rats with a value between 0.1 and 0.9 changed their classification in >10% of the bootstrap samples, and these animals were excluded from the experiment. Four animals of 42 were excluded based on this criterion.

### Experiment 1: Social defeat and prepro-orexin expression

In one cohort of either control rats or those exposed to 7-d social defeat, *in situ* hybridization was used to measure the level of prepro-orexin mRNA in the lateral hypothalamus. Briefly, 20-µm sections of the lateral hypothalamus from brains of control, passively coping, and actively coping rats were collected on a cryostat (rostral-caudal coordinates relative to bregma: –1.30 to –4.60 mm) and processed for *in situ* hybridization. Hybridization localization of mRNAs using ^35^S-labeled antisense mRNA probes was performed. In short, coronal brain slices encompassing the lateral hypothalamus were hybridized, *in situ*, with antisense to orexin (donated by Dr. Teresa Reyes, University of Cincinnati). Hybridizations for all slices were conducted in a single lot followed by analysis of the signal on x-ray film. Routine controls consisted of sense-strand probes labeled to similar specific activities as the antisense probes. X-ray films were analyzed using ImageJ. Background estimates were produced by optical density measurements over non–positively hybridized regions.

Another cohort of either control rats or those exposed to 7 d of social defeat was used to assess orexin mRNA by qPCR. Control, passively coping, and actively coping rats were killed and fresh punches of lateral hypothalamus were collected. RNA was extracted with Purelink mRNA kit according to the manufacturer’s protocol (Thermo Fisher Scientific). RNA was reverse transcribed to cDNA using a high-capacity cDNA reverse transcription kit (Thermo Fisher Scientific). qPCR was performed using Taqman Gene Expression Assays (Thermo Fisher Scientific) with primers for prepro-orexin (Hs01891339_s1) and Actb (Hs01060665_g1) and the Applied Biosystems 7500 Real Time PCR System.

### Experiment 2: Inhibiting orexins during social defeat using DREADDs

DREADDs are viruses that contain synthetic GPCRs and can be activated by the otherwise pharmacologically inert ligand clozapine-*N*-oxide (CNO). We obtained the CMV-hM4Di-mCitrine plasmid from Dr. Bryan Roth (University of North Carolina, Chapel Hill). Slice electrophysiology has demonstrated that CNO application to hippocampal cells expressing this Gi-coupled designer receptor causes hyperpolarization and decreased firing rate (Armbruster et al., 2007). Recent studies have found that CNO silencing of particular brain areas can produce striking behavioral effects, such as a reduction in anxiety-like behavior (McCall et al., 2015). We next obtained a 1295-bp promoter for human prepro-orexin gene (Ple112) from Addgene plasmid no. 29004 (gift of Dr. Elizabeth Simpson, University of British Columbia). This promoter was subcloned upstream of the hM4Di-mCitrine region to replace the construct’s CMV promoter to drive transgene expression specifically in orexin neurons. The fragment Ple112-hm4Di-mCitrine was then subcloned between the inverted terminal repeats (ITRs) of the AAV2 genome. In a separate study, we found that AAV1 serotype displayed optimal tropism for Sprague-Dawley rat hypothalamic neurons when delivered *in vivo* and compared to AAV5, 8, and 9 expression of GFP reporters driven by common constitutively active promoters Synapsin and CB7. Based on this finding, the University of Pennsylvania Viral Vector Core produced a recombinant adenovirus rAAV2/1-Ple112-hM4Di-mCitrine (using AAV1 serotype capsid for optimal transduction in orexin neurons) for our use. Previous studies using this virus demonstrate that activation of this construct *in vivo* decreases cFos expression in orexin neurons, supporting the efficacy of this construct (Grafe et al., 2017a).

**Figure 2.**
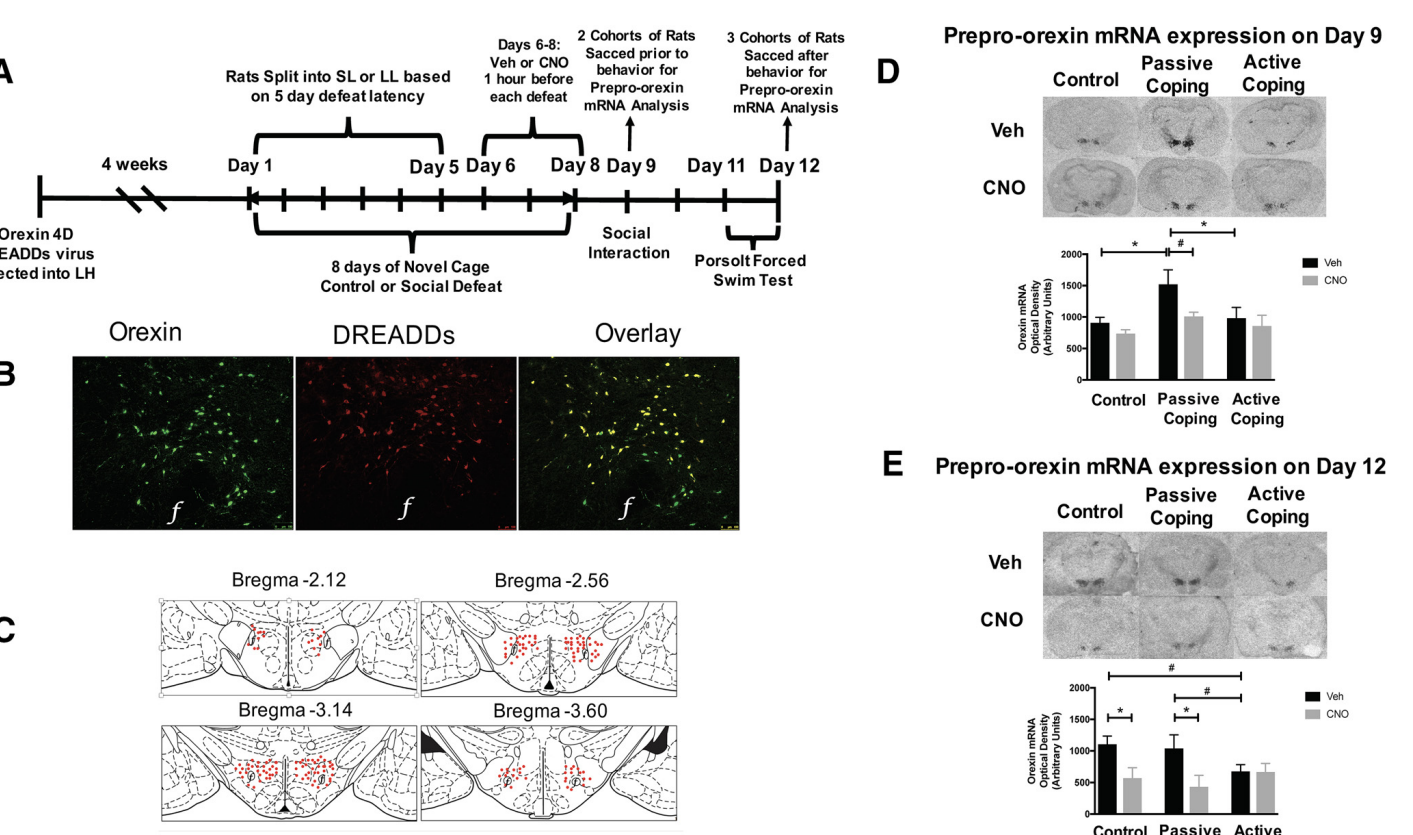
Expression of DREADDs-containing virus and inhibiting orexins during social defeat. ***A***, A timeline of the experimental paradigm. 4 wk after DREADDs injection, rats are exposed to 8 d of social defeat (the latter 3 d Veh or CNO is injected before defeat), followed by social interaction and forced swim test. ***B***, Representative images displaying viral expression of DREADDs in the lateral hypothalamus (LH) at 4 wk. ***C***, Composite image displaying the spread of viral expression along the LH is depicted using rat brain atlas images (Paxinos and Watson, 1998). Each red dot represents a cell expressing the viral tag. ***D***, Prepro-orexin expression in vehicle- and CNO-treated control, passive coping, and active coping rats on day 9 (before further behavioral testing). Top: representative images of *in situ* radiolabeling for vehicle- and CNO-treated prepro-orexin in control, passive coping, and active coping rats. Bottom: quantification of *in situ* radiolabeling in each treatment group reveals that actively coping rats have significantly less prepro-orexin mRNA in the lateral hypothalamus compared with passively coping rats. CNO treatment reduces prepro-orexin expression in passively coping rats to levels similar to that of actively coping rats. ***E***, Prepro-orexin expression in vehicle- and CNO-treated control, passive coping, and active coping rats on day 12 (after social interaction and forced swim test behaviors). Top: Representative images of *in situ* radiolabeling for vehicle- and CNO-treated prepro-orexin in control, passive coping, and active coping rats. Bottom: quantification of *in situ* radiolabeling in each treatment group reveals that actively coping rats have less prepro-orexin mRNA in the lateral hypothalamus compared with passively coping rats. CNO treatment reduces prepro-orexin expression in both control and passively coping rats to levels similar to that of actively coping rats. *, *p* < 0.05; ^#^, *p* < 0.10.

Five cohorts of male rats (20 rats per cohort) were anesthetized using a cocktail of ketamine, xylazine, and acepromazine. Using stereotaxic technique, virus containing the DREADDs construct (10^9^ titer, 1 µl bilaterally) was injected into the lateral hypothalamus (2.5 mm caudal to bregma, 1.8 mm from midline and 8 mm ventral). We verified the expression of the DREADDs constructs by immunofluorescence and determined that optimal expression occurs at 4 wk postinjection. Thus, virus was expressed for 4 wk before social defeat procedures, and subsequent behavior was assessed (see experimental paradigm in Fig. 2*A*).

Male rats expressing DREADDs-containing virus were either assigned to a control condition or exposed to the social defeat paradigm. Defeated rats were exposed to 5 d of social defeat without orexin manipulation, allowing the emergence of passively and actively coping phenotypes based on average defeat latency over those 5 d. In the original study describing these naturally occurring differences in response to social defeat (Wood et al., 2010), the 5-d latencies are predictive of the 7-d defeat latencies. In addition, previous publications have demonstrated that 5 d of social defeat is sufficient to induce depressive-like behavior in passively coping rats (but not actively coping rats; Wood et al., 2015; Finnell et al., 2017a). Thus, 5 d of defeat is comparable to the 7 d of defeat as performed in experiment 1. Body weights were collected both before and after the 5 d of defeat. Then control, actively coping, and passively coping rats were randomly assigned to a vehicle or CNO group. In these rats, on days 6–8, either vehicle (saline and 8% dimethyl sulfoxide) or CNO (2 mg/kg; Sigma-Aldrich) was injected 60 min before each defeat (or at the same time of day in control rats). This dose is in accordance with doses used in previous DREADDs studies in rats (Farrell and Roth, 2013) and those we have previously used to inhibit orexin neurons using hM4Di DREADDs. This timing was chosen because CNO promotes behavioral effects in the rat within 30 min of administration, and effects last up to 4 h after administration (Alexander et al., 2009; Farrell and Roth, 2013; Hasegawa et al., 2014). Thus, the final groups were vehicle-treated control rats, CNO-treated control rats, vehicle-treated passively coping rats, CNO-treated passively coping rats, vehicle-treated actively coping rats, and CNO-treated actively coping rats.

On day 9, two cohorts were sacrificed, and *in situ* hybridization was used to measure the level of prepro-orexin mRNA in the lateral hypothalamus, as described in experiment 1. Three other cohorts were exposed to a social interaction test in a 70 × 70-cm arena. In brief, rats were placed in the arena with another male Sprague-Dawley rat of similar size and weight. Rats were allowed to interact in this arena for 15 min and were videotaped and analyzed by Ethovision XT video tracking software (Noldus Information Technology). Latency to interact (time in seconds until experimental rat explores stimulus rat), total time interacting (number of seconds that the experimental rat explores stimulus rat), and distance moved were calculated. Total time interacting and latency to interact were verified by hand coding from an observer blind to experimental conditions.

On days 11 and 12, rats were tested in the Porsolt forced swim test (FST). Based on the work of Lucki (1997), FST was performed on two consecutive days. Rats were exposed to 15 min of forced swim (day 1), followed 24 h later by a 5-min forced swim (day 2). The 5-min swim test was videotaped from directly above the clear glass cylinder (46 cm height, 20 cm diameter), filled to 35 cm with water at a temperature of 25°C (±1°C). Two trained observers categorized the rat’s videotaped behavior (day 2) every 5 s for immobility, swimming, or climbing. Percentage time swimming and climbing were also combined to analyze percentage time active.

After all behavioral experiments were complete, animals were killed, brains were collected, and 20-µm lateral hypothalamic slices were analyzed for both prepro-orexin mRNA expression (as previously described) and viral expression. Specifically, immunofluorescence for visualizing the virus tag was conducted as follows. Tissue was incubated with primary antibodies for both Orexin A (1:250, sc-8070; Santa Cruz Biotechnology) and GFP (1:500, ab290; Abcam). As the mCitrine tag on the DREADDS virus originates from *Aequorea victoria* jellyfish, GFP antibodies are known to react with these proteins (Le et al., 2006). Sections were then incubated with Alexa Fluor 488 donkey anti-goat and Alexa Fluor 647 donkey anti-rabbit secondary antibodies (1:200, A-11055 and A-31573; Life Technologies). Images were acquired with a Leitz DMR microscope with a digital camera (Leica; Fig. 2*B*). The NIH ImageJ colocalization plugin was used to determine percentage orexin cells transduced by the virus. Approximately 70% of the orexin cells are transfected at this time, consistent with previous studies. The numbers of DREADDs-expressing cells and Orexin A–labeled cells were also methodically counted from anterior to posterior extent of the lateral hypothalamus (–2.12 to –3.60 mm; Fig. 2*C*).

### Statistical analysis

Data are presented as the mean ± SEM. For orexin expression by *in situ* and qPCR, and body weight gain, one-way ANOVA was performed, followed by Tukey’s *post hoc t* test. For orexin expression (before and after behavior), social interaction, and forced swim test data, two-way ANOVA [stress (control, passive coping, or active coping) by drug (vehicle or CNO) treatments] was used, followed by Tukey’s *post hoc t* tests. All analyses used α = 0.05 as the criterion level of significance. Statistical analysis was conducted with GraphPad Prism (GraphPad Software) to identify statistical differences. Superscript letters listed with *p*-values correspond to the statistical tests shown in Table 1.

**Table 1.** Statistical analysis

Location	Data structure	Type of test	Confidence interval (95%)
a	Normal distribution	*t* test	Passive vs. active latency: 189.8 to 284.3
b	Normal distribution	One-way ANOVA, Tukey’s *t* test	Control vs. active coping orexin mRNA: 1.3 to 2093; passive vs. active coping orexin mRNA: 749.8 to 3180
c	Normal distribution	Correlation	Latency vs. orexin mRNA: –0.8 to 0.0
d	Normal distribution	One-way ANOVA, Tukey’s *t* test	Control vs. active coping orexin mRNA: 0.2 to 1.2; passive vs. active coping orexin mRNA: 0.1 to 1.2
e	Normal distribution	Correlation	Latency vs. orexin mRNA: –0.8 to –0.0
f	Normal distribution	One-way ANOVA, Tukey’s *t* test	Control vs. passive coping body weight: 5.6 to 19.5; control vs. active coping body weight: 0.2 to 13.8
g	Normal distribution	Two-way ANOVA, Tukey’s *t* test	Vehicle-treated control vs. vehicle-treated passive coping orexin mRNA: –1157 to –73.81; vehicle-treated passive coping vs. vehicle-treated active coping orexin mRNA: 80.6 to 1002; vehicle-treated passive coping vs. CNO-treated passive coping orexin mRNA: –26.99 to 1057
h	Normal distribution	Two-way ANOVA, Tukey’s *t* test	Vehicle-treated passive coping vs. vehicle-treated active coping interaction time: –101.5 to –0.6; vehicle-treated passive coping vs. CNO-treated passive coping interaction time: –116.6 to –8.6
i	Normal distribution	Two-way ANOVA, Tukey’s *t* test	Vehicle-treated control vs. vehicle-treated passive coping % immobility: –26.4 to –3.9; vehicle-treated control vs. CNO-treated control % immobility: –25.7 to –5.6; vehicle-treated passive coping vs. CNO-treated passive coping % immobility: 0.6 to 23.7
j	Normal distribution	Two-way ANOVA, Tukey’s *t* test	Vehicle-treated control vs. vehicle-treated passive coping % activity: 3.9 to 26.4; vehicle-treated control vs. CNO-treated control % activity: 5.6 to 25.6; vehicle-treated passive coping vs. CNO-treated passive coping % activity: –23.7 to –0.6
k	Normal distribution	Two-way ANOVA, Tukey’s *t* test	Vehicle-treated control vs. vehicle-treated passive coping % swimming: 4.3 to 26.3; vehicle-treated control vs. CNO-treated control % swimming: 3.5 to 24.0
l	Normal distribution	*t* test	Vehicle vs. CNO-treated social interaction time: –32.5 to 50.8
m	Normal distribution	*t* test	Vehicle vs. CNO-treated social interaction latency: –24.8 to 38.8
n	Normal distribution	*t* test	Vehicle vs. CNO-treated social interaction distance traveled: –38,305 to 63,114
o	Normal distribution	*t* test	Vehicle vs. CNO-treated % immobility: –5.7 to 21.8
p	Normal distribution	*t* test	Vehicle vs. CNO-treated % activity: –21.8 to 5.7
q	Normal distribution	Two-way ANOVA, Tukey’s *t* test	Vehicle-treated control vs. vehicle-treated active coping orexin mRNA: –35.82 to 889.3; vehicle-treated passive coping vs. vehicle-treated active coping orexin mRNA: –121.5 to 846.1; vehicle-treated control vs. CNO-treated control orexin mRNA: 92.88 to 975.8; vehicle-treated passive coping vs. CNO-treated passive coping orexin mRNA: 123.8 to 1091

## Results

### Prepro-orexin expression in rats vulnerable or resilient to defeat

After 7 d of social defeat (Fig. 1*A*), rats were split into passive coping and active coping clusters based on average latency to defeat. Rats that displayed passive coping had an average defeat latency of 182 s, whereas rats that displayed active coping had an average defeat latency of 419 s (Fig. 1*B*; *p* < 0.001^a^, *t* test, *n* = 16/group). Prepro-orexin expression was then examined in two separate cohorts of control, passively coping, and actively coping rats; one cohort was used for *in situ* radiolabeling and the other was used for qPCR. Quantification of *in situ* radiolabeling in each treatment group revealed that actively coping rats had significantly less prepro-orexin mRNA in the lateral hypothalamus compared with passively coping rats (Fig. 1*C*, *F*(2,23) = 8.4, *p* = 0.002^b^; one-way ANOVA followed by Tukey’s *t* test; *n* = 8/group, 4 slices per animal). Moreover, there was a trend for a negative correlation between average defeat latency and prepro-orexin mRNA as measured by *in situ* radiolabeling (Fig. 1*C*, *R* ^2^ = 0.260, *p* = 0.062^c^). qPCR analysis of prepro-orexin levels in another cohort of rats demonstrated a consistent result: rats that displayed active coping expressed significantly less prepro-orexin mRNA than passively coping rats (Fig. 1*D*, *F*(2,22 = 4.4, *p* = 0.025^d,^ one-way ANOVA followed by Tukey’s *t* test; *n* = 8/group). Average defeat latency was negatively correlated with orexin mRNA as quantified by qPCR (*R* ^2^ = 0.276, *p* = 0.044^e^). Together, these results demonstrate that lower orexin expression is associated with an active coping strategy, and thus, based on previous findings (Wood et al., 2010), a resilient phenotype.

### Inhibition of orexins during the last 3 d of social defeat using DREADDs

To determine the effects of orexin inhibition on behavioral outcomes produced by social defeat, rats were first injected with an inhibitory DREADDs viral construct which was allowed to express for 4 wk (for experimental paradigm and confirmation of DREADDs expression, see Fig. 2*A–C*). Next, rats underwent social defeat for 5 d and were split into passive and active coping groups based on average defeat latency (see Methods for more detail on how this analysis was performed). As expected, body weight gain was significantly different between control and defeated groups (data not shown; *F*(2,38) = 6.766, *p* = 0.003^f^, one-way ANOVA followed by Tukey’s *t* test; *n* = 16/group). Specifically, control rats that did not undergo social defeat stress showed significantly more weight gain than those that did. In addition, rats that displayed passive coping had less weight gain compared with actively coping rats.

Two cohorts of rats were sacrificed before social interaction behavior to examine the effect of social defeat and vehicle or CNO treatment on prepro-orexin mRNA expression (Fig. 2*D*). Prepro-orexin mRNA levels appeared to differ between treatment groups (defeat effect, *F*(2,27) = 3.2, *p* = 0.055^g^; CNO effect, *F*(1,27) = 3.6, *p* = 0.067, two-way ANOVA, followed by *t* tests, *n* = 6/group). Particularly, vehicle-treated actively coping rats had lower levels of prepro-orexin mRNA than vehicle-treated passively coping rats. Thus, this phenotype of lower orexin expression in rats that demonstrate an active coping strategy is stable. Additionally, CNO treatment during the last 3 d of defeat reduced prepro-orexin mRNA levels in passive coping rats to that of control and active coping rats. To examine whether reducing orexins promotes behavioral correlates of resilience, 3 additional cohorts of rats were exposed to 5 d of social defeat followed by vehicle or CNO treatment before each defeat on days 6–8. These cohorts were then assayed for anxiety-like behavior in the social interaction test and depressive-like behavior in the forced swim test.

**Figure 3.**
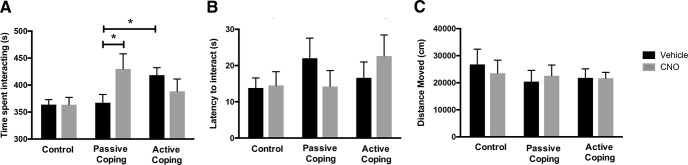
Social interaction behavior after the social defeat paradigm. ***A***, Time spent interacting with the stimulus rat. Actively coping rats spend significantly more time interacting than passively coping rats. CNO treatment (inhibition of orexin neurons) increases time spent interacting in passively coping rats. ***B***, Latency to interact with the stimulus rat. There were no significant differences in latency to interact between the treatment groups. ***C***, Distance moved in the social interaction arena. There were no significant differences in the distance moved between the treatment groups. *, *p* < 0.05.

On day 9 of the experimental paradigm, rats were tested for social interaction with a stimulus rat (Fig. 3). The amount of time spent interacting was significantly different between treatment groups (Fig. 3*A*; defeat effect, *F*(2,48) = 3.3, *p* = 0.043^h^; interaction effect, *F*(2,48) = 3.4, *p* = 0.045, two-way ANOVA followed by Tukey’s *t* test, *n* = 12/group). Importantly, in vehicle-injected groups, actively coping rats spent significantly more time interacting than passively coping rats, replicating previous findings (Chen et al., 2015b; Pearson-Leary et al., 2017). CNO treatment (inhibition of orexin neurons during the last 3 d of social defeat) increased time spent interacting in passively coping rats but had no effect in actively coping rats or control rats. There were no significant differences in latency to interact between the treatment groups (Fig. 3*B*). Additionally, there were no significant differences in the distance moved between the treatment group, indicating that the manipulation of orexin action did not simply change general arousal or locomotor activity (Fig. 3*C*). Thus, dampening of orexin action during the last 3 d of social defeat specifically increased social interaction in the vulnerable population of rats to the level of resilient rats.

**Figure 4.**
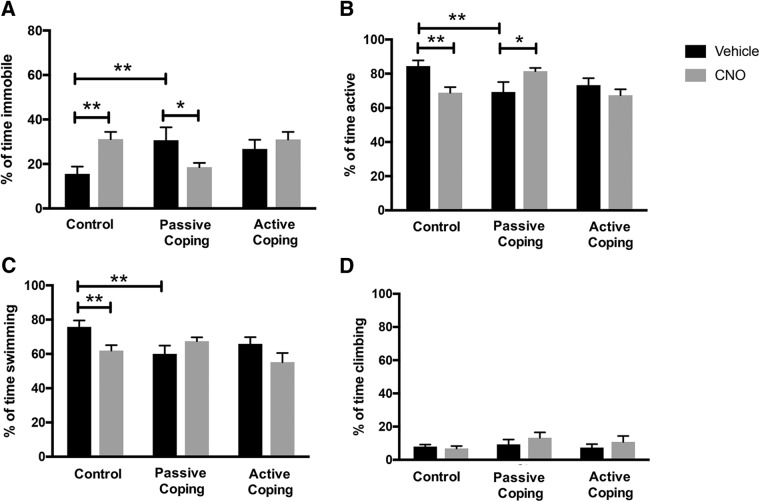
Forced swim test behavior after the social defeat paradigm. ***A***, Percentage of time spent immobile in the forced swim test. Passively coping rats spend significantly more time immobile than control rats. CNO treatment (inhibition of orexin neurons) decreases time spent immobile in passively coping rats. CNO treatment (inhibition of orexin neurons) increases time spent immobile in control rats. ***B***, Percentage of time spent active in the forced swim test. Vehicle-treated passively coping rats spend significantly less time active than control rats. While CNO treatment decreases time spent active in control rats, it increases time spent active in passively coping rats. ***C***, Percentage of time spent swimming in the forced swim test. Passively coping rats spend significantly less time swimming than control rats. CNO treatment decreases time spent swimming in control rats. ***D***, Percentage of time spent climbing in the forced swim test. There were no significant differences in time spent climbing between the treatment groups. *, *p* < 0.05; **, *p* < 0.01.

On days 11 and 12 of the experimental paradigm, rats were tested in the Porsolt forced swim paradigm to assess depressive-like behavior (Fig. 4). Analysis of percentage time immobile revealed significant differences between treatment groups (Fig. 4*A*; interaction effect, *F*(2,47) = 6.6, *p* = 0.003^i^, two-way ANOVA followed by Tukey’s *t* test, *n* = 12/group). Specifically, vehicle-treated passively coping rats spent significantly more time immobile than control rats, replicating previous findings (Wood et al., 2010). CNO treatment (inhibition of orexin neurons during the last 3 d of social defeat) decreased time spent immobile in passively coping rats but had no effect in actively coping rats. CNO treatment in control animals increased time spent immobile. Percentage time spent active was next analyzed, revealing that vehicle-injected passively coping rats spent less time active than control rats, and inhibition of orexin neurons during social defeat reversed this effect (Fig. 4*B*; interaction effect, *F*(2,46) = 6.7, *p* = 0.003^j^, two-way ANOVA followed by Tukey’s *t* test, *n* = 12/group). Activity was then subdivided into swimming and climbing behaviors. Analysis of swimming behavior revealed that vehicle-injected passively coping rats spent significantly less time swimming than vehicle-injected control rats (Fig. 4*C*; interaction effect, *F*(2,46)= 4.1, *p* = 0.022^k^, two-way ANOVA followed by Tukey’s *t* test, *n* = 12/group). Moreover, CNO treatment decreased time spent swimming in control rats but had no effect on passively or actively coping rats. Lastly, there were no significant differences between the treatment groups in climbing behavior (Fig. 4*D*). Thus, it appears that the differences in activity between treatment groups can mostly be attributed to swimming behavior. However, CNO treatment in passively coping rats appeared to increase a combination of both swimming and climbing; these measures were only significantly increased when summed together as total activity.

A control experiment was performed to determine if CNO alone had any impact on behavior in non–DREADDs-expressing rats. Specifically, a separate naive cohort of rats was injected with either vehicle or CNO for three consecutive days, followed by testing in the social interaction test and forced swim test. This was the same treatment regimen as in the original experiment above. The data indicate that total social interaction time did not differ between vehicle- and CNO-treated groups (395.6 ± 10.5 vs. 404.8 ± 16.5 s, *p* = 0.600^l^, *t* test, *n* = 6/group). Latency to interact was also not different between treatment groups (13.0 ± 2.9 vs. 19.4 ± 6.0 s, *p* = 0.534^m^, *t* test, *n* = 6/group). Moreover, distance traveled in the social interaction arena did not differ between treatment groups (21,458.2 ± 3872.1 vs. 28,746.6 ± 9193.6 cm, *p* = 0.403^n^, *t* test, *n* = 6/group). Lastly, neither percentage immobility nor total activity differed between vehicle- and CNO-treated groups in the forced swim test (immobility: 20.8 ± 2.5 vs. 28.9 ± 4.2, *p* = 0.19^o^, *t* test, *n* = 6/group; activity: 79.2 ± 4.2 vs. 71.1 ± 2.5, *p* = 0.19^p^, *t* test, *n* = 6/group). In summary, CNO treatment alone in non–DREADDs-expressing rats did not cause significant changes in the social interaction or forced swim test compared with vehicle-treated animals. These results indicate that the effects of DREADDs inhibition in the experiment above were not due to the effects of CNO alone.

Prepro-orexin mRNA levels differed between treatment groups after the forced swim test on day 12 (Fig. 2*E*, CNO effect, *F*(1,38) = 8.0, *p* = 0.007^q^, two-way ANOVA followed by Tukey’s *t* test, *n* = 12/group). Namely, vehicle-treated actively coping rats had lower prepro-orexin expression compared with vehicle-treated passively coping rats. Once again, this phenotype of lower orexin expression in actively coping rats is stable. CNO treatment reduced prepro-orexin expression in both control and passively coping rats. Overall, these results indicate that reducing orexin action in the last 3 d of social defeat increased social interaction time and reversed the depressive-like behavior observed in the vulnerable population of rats.

## Discussion

These experiments used a social defeat paradigm that generates two different populations of rats that demonstrate either passive or active coping strategies, based on their average latency to be defeated. Previous studies have indicated that rats displaying a passive coping strategy demonstrate subsequent anxiety- and depressive-like behaviors (Wood et al., 2010; Chen et al., 2015b; Pearson-Leary et al., 2017). This is consistent with human studies, which have demonstrated that passive coping is more often associated with the development of major depressive disorder (MDD). Our results suggest a substrate of resilience, namely, the neuropeptides orexins, which are known to mediate the stress response and are altered in patients with MDD and PTSD (Kuru et al., 2000; Yehuda, 2000; Winsky-Sommerer et al., 2004; Spinazzi et al., 2006; Furlong et al., 2009; Strawn et al., 2010; Chen et al., 2015a). In short, we first discovered that lower orexin expression was associated with active coping strategies. We next inhibited orexin action during the last 3 d of social defeat, and this produced an increase in social interaction and a decrease in depressive-like behaviors in passively coping rats. Thus, we established that low orexin function contributes to the active/resilient behavioral phenotypes in response to repeated social defeat stress.

Both *in situ* radiolabeling and qPCR approaches revealed that rats displaying active coping (resilient rats) expressed significantly lower levels of prepro-orexin mRNA compared with rats that displaying passive coping (vulnerable rats) as well as control rats. This led to the hypothesis that it is the lower levels of orexins that underlie resilience to repeated social stress. To test this, rats first underwent 5 d of social defeat stress to establish active and passive coping phenotypes. Next, to determine whether reducing orexins promotes resilience, we inhibited orexin neurons before each social defeat for three additional days using DREADDs. Importantly, we found that CNO treatment during the last 3 d of defeat reduced prepro-orexin mRNA levels in passive coping rats to that of active coping rats. Additionally, inhibition of orexin neurons before each defeat resulted in increased social interaction behavior and decreased immobility during forced swim test in passively coping (vulnerable rats) to the level of actively coping (resilient rats). As expected, inhibition of orexin neurons before each defeat had no effect on subsequent behavior in resilient rats, as our experiments indicate they already express very low levels of orexins. Thus, inhibiting orexin action during the last 3 d of social defeat increased social interaction and decreased depressive-like behaviors specifically in the vulnerable population of rats, thereby promoting resilience. This indicates that dampened orexin function under conditions of stress contributes to resilience to social defeat.

Prepro-orexin expression was lower in resilient rats compared with vulnerable rats by both *in situ* radiolabeling and qPCR methods. This phenotype was stable in multiple cohorts of rats, immediately after repeated social defeat, as well as after several additional behavioral tests. We cannot determine if prepro-orexin expression was lower in resilient rats before social stress or as a consequence of social stress; thus, it is possible it is a pre-existing difference. A previous study demonstrated that control female rats had higher orexin expression than male rats, and as a result, females had persistent HPA activation in response to repeated restraint stress and were not able to habituate as fully as males (Grafe et al., 2017a). Just as females had higher levels of orexins before the stressor, and thus were inherently different from males, perhaps actively coping (resilient) rats are inherently different from passively coping (vulnerable) rats before social defeat occurs. In this respect, pre-existing differences in orexin expression impact future responses to stress. On the other hand, perhaps orexin function is also decreased with repeated exposure to social defeat in rats that become resilient to defeat; this is known to occur after repeated restraint stress (Grafe et al., 2017b). Currently, we are only able to measure cerebrospinal fluid levels of orexins terminally, and plasma levels of orexins are not a reliable indication of central orexin activity; thus, we cannot directly determine whether this difference in orexin expression predates exposure to stress.

Based on these data, we hypothesized that lower levels of orexin contribute to resilience to repeated social stress. After rats established active and passive coping phenotypes, orexins were inhibited by DREADDs in subsequent days of social defeat. Behavior was examined in both the social interaction and forced swim tests to determine how orexin action during defeat affects subsequent anxiety-like and depressive-like behaviors. Our results first demonstrated that vehicle-treated actively coping rats spent more time socially interacting than passively coping rats, replicating a previous finding (Wood et al., 2010). Orexin inhibition during the last 3 d of defeat increased the amount of time passively coping rats spent interacting with a stimulus rat, with total interaction time at a level comparable to that of actively coping rats. This result is consistent with earlier studies in which central injections of orexins produced anxiety-like behaviors in the light-dark test and elevated plus maze (Suzuki et al., 2005; Li et al., 2010; Avolio et al., 2011). As expected, inhibition of orexins in resilient rats during social defeat did not further increase their social interaction time, likely because resilient rats already express low levels of orexins. In sum, orexins promote anxiety behaviors, and dampening orexin action throughout repeated stress allows rats that are initially vulnerable to exhibit the resilient phenotype.

The increase in social interaction observed in CNO-treated passively coping rats was independent of the total amount of movement in the social interaction chamber. As orexins have been shown to modulate spontaneous physical activity (Kotz et al., 2002), it is important to note that the increase in social interaction was not accompanied by an increase in activity; thus, it is a socially specific behavioral result and not an effect on global arousal. Moreover, the orexin manipulation took place only during the last 3 d of social defeat, and not during this social interaction test; thus, short-term action of CNO treatment did not confound our results. However, our data indicate that CNO treatment on days 6–8 of social defeat can cause long-lasting changes in prepro-orexin mRNA expression, which then leads to changes in social interaction behavior.

In the FST, vehicle-treated passively coping rats spent significantly more time immobile than control rats, replicating a previous finding that passive coping during social defeat leads to depressive-like behavior (Wood et al., 2010). DREADDs-mediated inhibition of orexin neurons before three social defeat exposures reduced percentage time spent immobile in the vulnerable rats. Hence, increased orexin action may contribute to depressive-like behavior. However, we found that repeated CNO treatment increased immobility in control, nonstressed animals. Thus, inhibiting orexins in nonstress conditions increases depressive-like behavior. Our data show that prepro-orexin expression does not differ between vehicle- and CNO-treated control (nonstressed) animals, so this cannot explain the differences in percentage immobility in the FST. It is possible that other measures of orexin function, such as neuronal activation, may differ between vehicle- and CNO-treated control animals, explaining these differences in behavior. Ultimately, the effect of inhibiting orexins on immobility is dependent on whether the animal is stressed; different brain circuits involving orexins may be activated in these different conditions, explaining the opposing behaviors.

The link between the orexinergic system and depression remains equivocal, as clinical models report conflicting results. Specifically, different studies indicate that either hypoactivity (Brundin et al., 2007; Ito et al., 2008) or hyperactivity (Salomon et al., 2003; von der Goltz et al., 2011) of the orexinergic system is associated with MDD (Brundin et al., 2007; Lutter et al., 2008). Some inconsistencies may result from limitation of methods, as orexin levels in plasma are close to the resolving limit of radioimmunoassay (Chen et al., 2015a). Moreover, whether measures of orexin A in plasma or cerebrospinal fluid are physiologically meaningful and can act as proxy for orexin system activity remains to be established. However, a recent preclinical study provided a causal link between orexins and depressive-like behavior: pharmacological blockade of the orexin system during unpredictable chronic mild stress reduced subsequent immobility in the tail suspension test (Nollet et al., 2012). This result is consistent with our findings in that inhibition of orexins during stress decreases subsequent depressive-like behavior. As expected, inhibition of orexins in actively coping rats during the last 3 d of social defeat did not further decrease immobility, as our results indicate that actively coping (resilient) rats already express very low levels of orexins. Together, these data suggest that low levels of orexins may be a biomarker to predict resilience to stress and thus, a lower likelihood of developing depression.

Rats that displayed passive coping spent less time active (mostly due to a decrease in swimming) in the FST than control rats. However, blocking orexin action before three social defeat exposures appeared to increase both swimming and climbing in passively coping rats. Independently, these two measures were not significantly increased with CNO treatment. Specifically, CNO treatment increased the sum of these two behaviors together, measured as total activity. Swimming and climbing are known to be mediated by serotonin and norepinephrine, respectively, as antidepressants targeting these neurotransmitters can selectively increase these behaviors (Bogdanova et al., 2013). Indeed, it is known that orexins have direct connections with both serotoninergic and noradrenergic neurons to regulate sleep/wakefulness, thus, it makes sense that manipulating orexins may affect swimming and climbing behavior (Tabuchi et al., 2013; Zitnik, 2016). However, the effect of manipulating orexins on these behaviors appears to be dependent on whether the animal is stressed: inhibiting orexins in a control animal decreases activity (and increases immobility), whereas inhibiting orexins in a socially defeated rat increases activity (and decreases immobility). Thus, repeated stress must change the way these neurotransmitters interact with orexins.

Recent findings have indicated that a high dose (10 mg/kg) of CNO may allow for nonspecific effects of the metabolite clozapine on behavior (Gomez et al., 2017). Particularly, converted clozapine could have effects on the DREADDs or, if the levels are high enough, on endogenous clozapine binding sites as well. Moreover, another study found that 5-mg/kg doses of CNO have behavioral effects in Long-Evans rats not expressing DREADDs (MacLaren et al., 2016). We tested whether the lower dose of CNO (2 mg/kg) used in the present studies had nonspecific behavioral effects. We found that CNO treatment in non–DREADDs-expressing rats did not cause significant effects in the social interaction test or Porsolt forced swim test. Thus, we can conclude that the effects of DREADDs we observed in our studies were not due to actions of CNO or its metabolites but to DREADDs-induced inhibition of orexins.

The brain regions in which orexins act during stress to regulate subsequent anxiety-like and depressive-like behaviors are not fully elucidated. However, there are many key brain areas that likely play a role. For example, orexins have dense projections to brain areas relevant to anxiety- and depressive-like behaviors such as the paraventricular nucleus of the thalamus (PVT), locus coeruleus, prefrontal cortex, dorsal raphe, hippocampus, and amygdala (Peyron et al., 1998). Previous studies have demonstrated that orexins act in the PVT to induce anxiety-like behavior (Li et al., 2010; Heydendael et al., 2011, 2013). Another study found that orexin 1 receptors in the amygdala regulate stress-induced depressive-like behavior (Arendt et al., 2013). Other experiments indicate that orexin interaction with the dorsal raphe may be important for regulation of stress-induced depressive-like behavior (Brown et al., 2001; Muraki et al., 2004). Future studies should further examine the role of specific brain regions where orexins may be acting to promote resilience and identify genes mediating these orexin effects.

Because orexins are known to underlie arousal and appetite, it is possible that inhibition of these neuropeptides with DREADDs affected these physiologic parameters and, thus, may have influenced our results (Sakurai, 2014). For example, if inhibiting orexin action allowed animals to sleep more, perhaps this could have subsequently decreased anxiety-like or depressive-like behavior. While our results show that there were no changes in general arousal between treatment groups in terms of total movement during behavioral tests, measuring sleep parameters after orexin manipulation may provide more insight. Additionally, though we did not measure food intake throughout the study, there were no differences in body weight gain between vehicle- and CNO-treated rats, indicating that changes in appetite and food intake did not have a significant effect on the present results.

The results from this study demonstrate that orexin expression is lower in rats resilient to social defeat stress. To provide a causal link between decreased orexins and resilience, we inhibited orexins during the last 3 d of social defeat stress and reversed the negative behavioral effects of social defeat in previously vulnerable rats. These findings highlight orexins as previously uncharacterized substrates of resilience.

## Synthesis

Reviewing Editor: Karen Szumlinski, University of California at Santa Barbara Decisions are customarily a result of the Reviewing Editor and the peer reviewers coming together and discussing their recommendations until a consensus is reached. When revisions are invited, a fact-based synthesis statement explaining their decision and outlining what is needed to prepare a revision will be listed below. The following reviewer(s) agreed to reveal their identity: Jacob Raber, Rodrigo España.

Synthesis: Having discussed your report with 2 expert reviewers in the field, I have reached the decision of ‘Revise and Rereview’ as we all concur that the studies described in your report have the potential to advance our knowledge pertaining to the role for orexin in regulating behavior following social defeat. The reviewers and I agree that the report could be much improved by a greater discussion of the observed effects of the CNO treatment, within the context of the findings published recently in both eNeuro and Science indicating behavioral effects of CNO. Further, the reviewers also concurred that mRNA measurements for both the peptide and receptor should be included in the revised article as such data would inform as to how your DREADD approaches impact orexin neurotransmission. Please find the specific comments of each individual reviewer below:

Reviewer 1

This is an important and timely contribution. In general, the experiments are straight forward and well performed. Two revisions are requested:

1. Analysis of the orexin mRNA levels in the animals following the distinct treatments. This is especially important as CNO causes the opposite effects in control animals. So one question is why. Based on the data shown, the orexin levels are pretty comparable in control and passive coping animals. The level of the outcome measures shown seem different in the two groups. That might be important as one wonders whether baseline measures might modulate the directions of effects of CNO that are not via DREADDs or whether CNO has two distinct effects in the passive coping animals and that the effect mediated via the DREADD is simply stronger.

2. The authors do not really discuss the effects seen with CNO in the control animals. However, especially in the context of the recent paper in Science from an intramural NIDA group about CNO effects and DREADD binding this will be important to elaborate on. Along the same lines, it would be safer to state in the discussion what CNO treatment did that consistently to refer to orexin reduction, even once these data are included. My recommendation would be to describe the potential non target effects of CNO. Again, for this study it might be not that critical as the direction of change in the control animals is in the opposite direction.

Reviewer 2

The present manuscript investigated whether orexin neuropeptides participate in resilience/vulnerability to a social defeat stress. The investigators used in situ hybridization and qPCR to demonstrate that actively coping rats expressed significantly lower orexin mRNA relative to passively coping rats and control rats. To examine whether decreasing orexin activity promotes resilience to social defeat stress, the investigators then used Gi DREADDs to selectively inhibit orexin neurons. Obtained results showed that inhibition of orexin neurons reduces anxiety- and depressive-like behavior in the passively coping rats. Overall this is a clearly-written manuscript and the results are interesting and timely. The methods are appropriate and experiments are generally well controlled. Concerns are detailed below.

1) The authors state that “inhibiting orexins throughout social stress decreased the socially relevant anxiety-like behavior”. There are a couple of issues with this statement. First, according to figure 3A, passive coping rats do not show anxiety-like behavior as indicated by similar levels of social interaction as control rats. Thus, the idea that there is anxiety that was decreased in these animals following orexin inhibition is unfounded. If anything it is the active coping rats that show aberrant social interaction relative to controls. Second, the statement indicates that orexin neurons were inhibited throughout the social defeat, but in fact this was only the case for the last three days. This should be made clear in a few places in the paper.

2) It appears that for the in situ and qPCR experiments rats were exposed to 7 days of social defeat and average defeat latencies over the 7 days were used to determine passive vs active coping. By comparison, in the DREADDs experiments, rats were exposed to 5 days of social defeat to obtain average defeat latencies. Is there evidence that the 5 day average can predict the 7 day average? In other words, do the investigators have evidence that just 5 days of social defeat produces the same behavioral effects as 7 days. It is possible that orexin neuron inhibition is merely mitigating the effects of the remaining 3 social defeats - effectively making the model a 5 day social defeat model) rather than modifying the effects of the previous 5 social defeats as suggested in the current manuscript. A control experiment in which only 5 days of social defeat also produced similar behavioral effects as the 7 day model would easily resolve this possibility. Also, was there any difference in defeat latencies from the remaining 3 days of social defeat in rats with orexin neuron inhibition?

3) The link provided for the cluster analysis code was broken. Moreover, it would be useful if the authors provided more detail as to how the cluster analysis was done and how it led to splitting into passive and active coping rats. Related to this, it is important for the authors to report the number of animals that were dropped from analysis due to falling within the 0.1 - 0.99 range.

4) The authors should report on the number/percentage of GFP labeled neurons that were not orexin positive. This will give an indication of how selective the DREADDs virus was for orexin neurons.

5) It appears from the methods that the authors did not include a CNO alone control for their experiments. The authors should include a discussion of the possibility that some of their effects may be associated with non-specific CNO effects. This is especially important due to the recent observation by Gomez et al., 2017 (Science) showing that CNO's effects are likely related to conversion to clozapine which is known to have behavioral effects on its own.

6) The authors do not provide an interpretation of why there is increased immobility in the forced swim test for control rats treated with CNO? This should be discussed in relation to the effects observed in stressed rats.

7) The idea that reduced orexin activity promotes resilience and that control and vulnerable rats have normal/equal orexin activity is very interesting. Whether this is a pre-existing state for these animals or whether it is unveiled by stress is unclear as the authors point out themselves. Based on the spread of data in the control group and the relative similarity in orexin levels to vulnerable rats, can the authors speculate on whether they believe there is a preexisting orexin state that dictates resilience? In other words, if the control rats had been stressed would they show a similar spread / clustering of behavior or orexin mRNA? I recognize that this may be a challenging and overly speculative topic to discuss, but it would be a nice touch if it could be accomplished.

8) I suggest that the figure panels in figure 4 be reordered in this way. (A) immobility - (B) % time Active - (C) % time swimming - (D) % time climbing. I believe this would allow the investigators to compare immobility with activity and then show more in-depth analysis by splitting activity into swimming and climbing.

9) The paper is well-written but there are a couple of typos.

Page 11 line 357 passively coping rats spent less time active that control rats

Wood et al., 2010 is duplicated
